# Low-Intensity Pulsed Ultrasound-Mediated Blood-Brain Barrier Opening Increases Anti-Programmed Death-Ligand 1 Delivery and Efficacy in Gl261 Mouse Model

**DOI:** 10.3390/pharmaceutics15020455

**Published:** 2023-01-30

**Authors:** Mohammed H. Ahmed, Isaias Hernández-Verdin, Emie Quissac, Nolwenn Lemaire, Coralie Guerin, Lea Guyonnet, Noël Zahr, Laura Mouton, Mathieu Santin, Alexandra Petiet, Charlotte Schmitt, Guillaume Bouchoux, Michael Canney, Marc Sanson, Maïté Verreault, Alexandre Carpentier, Ahmed Idbaih

**Affiliations:** 1 Institut du Cerveau—Paris Brain Institute—ICM, Inserm, CNRS, AP-HP, Hôpital de la Pitié Salpêtrière, Sorbonne Université, F-75013 Paris, France; 2 School of Cancer & Pharmaceutical Sciences, King’s College London, London SE1 9NH, UK; 3 Cytometry Department, Institute Curie, F-75006 Paris, France; 4 Pharmacokinetics and Therapeutic Drug Monitoring Unit, Inserm, CIC-1901, UMR ICAN 1166, AP-HP, Hôpital de la Pitié Salpêtrière, Sorbonne Université, F-75013 Paris, France; 5 CarThera, Institut du Cerveau et de la Moelle Épinière (ICM), F-75013 Paris, France; 6 Institut du Cerveau—Paris Brain Institute—ICM, Inserm, CNRS, AP-HP, DMU Neurosciences, Service de Neurologie 2-Mazarin, Hôpital de la Pitié Salpêtrière, Sorbonne Université, F-75013 Paris, France

**Keywords:** SonoCloud, GL261 mouse model, ultrasound-mediated drug delivery, glioblastoma, immune checkpoint inhibitors

## Abstract

Therapeutic antibodies targeting immune checkpoints have shown limited efficacy in clinical trials in glioblastoma (GBM) patients. Ultrasound-mediated blood–brain barrier opening (UMBO) using low-intensity pulsed ultrasound improved drug delivery to the brain. We explored the safety and the efficacy of UMBO plus immune checkpoint inhibitors in preclinical models of GBM. A blood–brain barrier (BBB) opening was performed using a 1 MHz preclinical ultrasound system in combination with 10 µL/g microbubbles. Brain penetration of immune checkpoint inhibitors was determined, and immune cell populations were evaluated using flow cytometry. The impact of repeated treatments on survival was determined. In syngeneic GL261-bearing immunocompetent mice, we showed that UMBO safely and repeatedly opened the BBB. BBB opening was confirmed visually and microscopically using Evans blue dye and magnetic resonance imaging. UMBO plus anti-PDL-1 was associated with a significant improvement of overall survival compared to anti-PD-L1 alone. Using mass spectroscopy, we showed that the penetration of therapeutic antibodies can be increased when delivered intravenously compared to non-sonicated brains. Furthermore, we observed an enhancement of activated microglia percentage when combined with anti-PD-L1. Here, we report that the combination of UMBO and anti-PD-L1 dramatically increases GL261-bearing mice’s survival compared to their counterparts treated with anti-PD-L1 alone. Our study highlights the BBB as a limitation to overcome in order to increase the efficacy of anti-PD-L1 in GBM and supports clinical trials combining UMBO and in GBM patients.

## 1. Introduction

Glioblastoma (GBM) is the most malignant primary brain tumor in adults, with a median overall survival of less than 18 months after initial diagnosis [1]. Despite significant efforts in the neuro-oncology field to develop new therapeutic alternatives, temozolomide (approved in 2005) remains the gold standard chemotherapy in GBM treatment [2]. For over five decades, research has been focused on developing new anti-cancer therapies for GBM, including anti-neoplastic agents [3], molecular targeted drugs [4], immunotherapeutic approaches [5], and angiogenesis inhibiting compounds [6]; however, the prognosis of patients has hardly improved [7]. Blood-brain barrier (BBB), which is specific to the blood vessels in the central nervous system (CNS), prevents most systemic therapeutic compounds from reaching the brain parenchyma and GBM cells [8] although it is disrupted in some areas (i.e., blood–tumor barrier).

Several innovative strategies have been studied to enhance the delivery of chemotherapeutic agents and antibodies to the brain [8]. Ultrasound mediated BBB opening (UMBO) using low-intensity pulsed ultrasound (LIPU) has now been studied in preclinical [9] and clinical settings [10]. LIPU is delivered to the brain simultaneously with an I.V. of micron-sized bubbles for a few minutes, allowing the microbubbles to oscillate. Microbubble oscillation produces mechanical stretching on vessel walls that allows a transient BBB opening [11]. UMBO has shown a good safety profile for BBB opening in recurrent GBM patients [10,12] and is now being studied in dozens of clinical trials using a range of transcranial [13] or implantable ultrasound devices [10] for treating both primary and secondary brain tumors as well as neurodegenerative diseases [14].

The choice of therapeutic agents to deliver after UMBO is crucial and remains a point of discussion among researchers. Direct stimulation of the immune system with immune checkpoint inhibitors (ICI, e.g., PD-1/PD-L1) showed promising effects alone or with other chemotherapies in multiple cancers. PD-L1 proteins are expressed as surface molecules by cancerous cells, such as GBM cells [15], and provide a tumor escape mechanism when bound to PD-1 proteins at the surface of activated T-lymphocytes leading to their exhaustion [16]. Despite their promise in other cancers, nivolumab (anti-PD-1) has shown no additional efficacy over bevacizumab in phase III clinical trials in recurrent GBM patients [17]. Similarly, Avelumab (anti-PD-L1) in combination with molecular targeted drugs did not improve the outcome of GBM patients [18]. In the present study, we evaluated the effect of anti-PD-L1 and anti-CTLA-4 alone and in combination with UMBO in syngeneic Gl261 mouse models.

## 2. Materials and Methods

### 2.1. Low-Intensity Pulsed Ultrasound Preclinical Device

The pre-clinical ultrasound system (CarThera, Paris, France) was identical to that described in other studies (Supplementary Material, Figure S1A) [9].The system consisted of a 1 MHz, 10-mm diameter acoustic transducer that was coupled to the head of the mouse at 15-mm from the transducer. Sonication was performed for 120 s using a 25,000-cycle burst at a 1 Hz pulse repetition frequency and an acoustic pressure of 0.3 MPa as measured in water.

### 2.2. Calibration of Low-Intensity Pulsed Ultrasound Device

**Figure 1:**
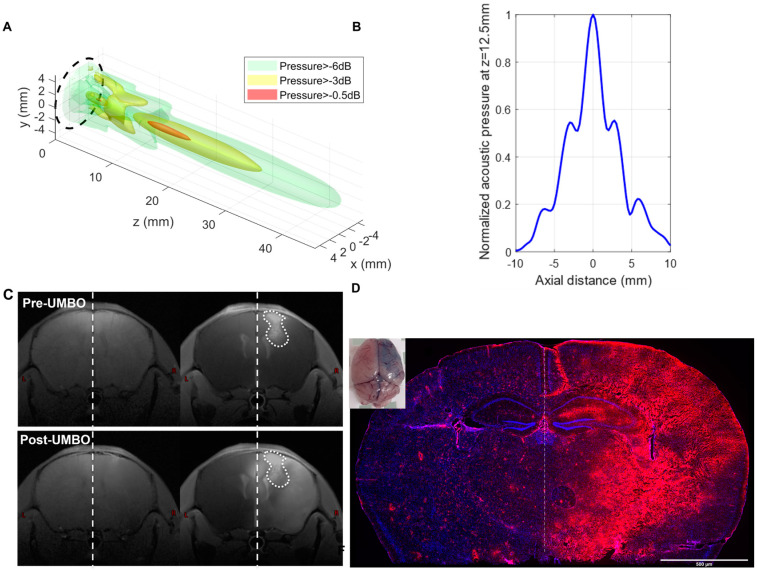
UMBO parameters are effective for BBB opening in healthy and Gl261 bearing mice. (Panel **A**,**B**): 3D structure of the ultrasound waves generated by the calibrated low-intensity ultrasound system. (Panel **C**): T1W MRI showed a marked Gd contrast enhancement within an hour following UMBO. An enhancement of Gd following UMBO in mice (*n* = 2, analysis of the Gd enhancement is shown in Supplementary Material, Figure S1C). The two top T1-MRI images were obtained before UMBO (pre-Gd left MRIs; and post-Gd). The two bottom T1-MRI images were obtained after UMBO (pre-Gd left MRIs; and post-Gd). UMBO is effective in C57BL/6 mice. right MRIs). (Panel **D**): Evans blue staining was enhanced in sonicated brain hemisphere compared to the control hemisphere visually and by fluorescence ((Panel **D**); Evans blue in red, DAPI in blue).

The ultrasound transducer (CarThera^®^) used in this study was calibrated on a regular basis (Figure 1A,B). The aim was to map the ultrasound field and to determine the electrical set point that the generator uses during the experiments to obtain the targeted acoustic pressure in situ. The calibration was performed using degassed water at room temperature with a 200 µm needle hydrophone (HNC0200, ONDA). A 2D acoustic field was scanned at 5 mm from the transducer surface with a 3-axis computer-controlled motorized positioning system (UMS, Precision Acoustics, UK). The 3D acoustic field was computed from this pattern with the Rayleigh integral. The hydrophone was positioned at the spatial peak pressure determined from the 3D acoustic field. The ratio between the active electrical power drawn by the transducer measured with an oscilloscope and the square of the spatial peak acoustic pressure was measured. This ratio is used as a calibration coefficient by the generator during subsequent experiments: the active electrical power needed to obtain the targeted pressure is calculated by the generator using this coefficient at the beginning of manipulation, and the generator adjusts its set point to obtain the specified active electrical power measured internally.

### 2.3. Ultrasound-Mediated Blood–Brain Barrier Opening (UMBO)

UMBO was delivered to both UMBO and UMBO plus anti-PD-L1 groups. Anti-PD-L1 (6E11 Genentech) was administered intraperitoneally in two independent experiments (*n* = 8) at a dose of 200 µg sixty minutes before UMBO application. Mice were maintained under anesthesia with isoflurane (2%, 2 L/min O_2_). For each UMBO application, 10 mL/kg SonoVue^®^ was injected through the intravenous route less than 10 s before the start of the ultrasound application. For each session, UMBO was validated using an additional control mouse. Each control mouse was injected intravenously with a solution of 2.7% Evans blue (Sigma, E2129) in phosphate buffer saline (PBS) at a dose of 4 mL/kg ten minutes post-sonication. All mice received 10 mL/kg warm saline injection in each treatment protocol before anesthesia to prevent any possible hypovolemia or hypothermia effect. I.P injection of anti-PD-L1 injection was given 60 min before sonication to ensure anti-PD-L1 absorption (Supplementary Material, Figure S1B). UMBO test mice were sacrificed 15 min following Evans’ blue injection, and their brain was harvested. The passage of Evans blue was assessed both visually and by ZEISS Axio-Scan fluorescence imaging of cryo-sectioned brains.

### 2.4. Cell Culture and In Vivo Studies

GL261 cells were cultured in Dulbecco’s modified essential medium (DMEM) supplemented with 10% fetal bovine serum and 1% penicillin/streptomycin. Cells were passaged twice weekly according to their confluence. The animal ethics committee at the Ministry of Higher Education and Research in Paris approved all protocols involving live mice (protocol #17503 and #26137). C57BL/6 mice were purchased from Charles River and were given a week of acclimation before starting any experiment.

GL261 was transduced with a *luciferase/mKate2* vector as described before [19]. GL261-luciferase (1.4 × 10^5^ cells/2 µL) were inoculated into the right caudate nucleus-putamen (AP +1 mm, DV −0.25 mm, ML −0.15 mm) of 7–8 weeks old C57BL/6 females using a stereotactic injection frame (David Kopf Instruments, Angeles, CA, USA). Mice were imaged using the IVIS Spectrum (PerkinElmer, Waltham, MA, USA) 10 min following a 2 mg subcutaneous injection of luciferin (Sigma Aldrich (MO, USA), L9504). The growth of GL261-luciferase cells was confirmed by two IVIS imaging one week apart of intracranial cell injection. We observed that mice with bioluminescence values lower than 5 × 10^5^ photon/second would not develop GBM tumors during the characterization of tumor growth in our mouse models. Therefore, we have included mice with bioluminescence values over 5 × 10^5^ photon/second. Mice were randomly placed into treatment arms once they passed the bioluminescence cutoff value.

Animals were treated with 200 µg of anti-CTLA-4 (Bristol-Myers Squibb, New York, NY, USA, G1-XAS-Ab), anti-PD-L1 (Genentech, San Francisco, CA, USA, 6E11), IgG1 (BXCELL, West Lebanon, NH, USA, BE0083), and InVivoPure pH 6.5 Dilution Buffer (BXCELL, IP0065) for four doses (Supplementary Material, Figure S1B). Unless stated otherwise, animals were sacrificed when they showed signs of tumor-associated illness (20% body weight loss or changes in behavior or posture).

### 2.5. Pharmacokinetic (PK) Analysis of Therapeutic Antibodies with and without UMBO

The PK analysis was performed using an identical molecular weight with similar conformational structure IgG1 isoform. Thirty-six mice were used in the pharmacokinetic experiment. Mice were separated into control and UMBO groups. Six time points were selected as follows: 0.15, 0.3, 3, 6, 24, 48, and 96 h. Each mouse received a 200 µg of nivolumab (Bristol-Meyers Squibb, New York, NY, USA) I.V. injection 10 min following the BBB opening. 100 μL of blood was collected through cardiac puncture using a pre-heparinized syringe. Serum was collected by centrifugation of the blood at 3500 rpm for 10 min. All samples (plasma and brain) were then analyzed using an ultra-performance liquid chromatography (UPLC) system coupled to mass spectrometry (LC-MS/MS; MS-8060, Shimadzu, Nakagyo-ku, Kyoto, Japan). Peak integration and quantification were performed using LabSolutions Insight LC-MS software. Nivolumab was quantified with signature peptide ASGGITFSNSGMHWVR by nano-surface and molecular orientation limited proteolysis (Shimadzu, Japan) [20].

### 2.6. MRI Data Acquisition

Two GL261-bearing mice were used in the experiment. Two sessions per mouse were completed in 2 consecutive days to decrease any distress effect of long isoflurane exposure. MRI acquisitions were performed using a preclinical 11.7 T MRI scanner (Biospec, Bruker BioSpin, Germany) equipped with a CryoProbe dedicated to mouse brain imaging (Biospec, Bruker BioSpin, Ettlingen, Germany). Gd enhancement volume was estimated on T_1_w MRI pre and post BBB opening manually on FSLeyes. Animals were anesthetized with 1% isoflurane in O_2_ (2 L/min). Respiratory rate and body temperature were monitored while mice were restrained. For each animal, the protocol consisted of: (i) acquiring pre-gadolinium enhancement anatomical T_1_-weighted (T_1_w) images using a multi-slice multi echo (MSME) sequence with the following parameters: repetition time (T.R.) = 400 ms, echo time (T.E.) = 5 ms (one single echo), four averages, 14 slices, and resolution = 60 × 60 × 500 μm^3^, (ii) following injection of a total volume of 100 µL of gadolinium (Gd; DOTAREM^®^,Guerbet, Aulnay-sous-Bois, France) at 0.5 mM and at physiological temperature in the tail vein of the mouse outside the MRI scanner, and (iii) acquiring post-gadolinium T_1_w images using the same sequence. The MGE sequence was acquired with the following parameters: T.R. = 80 ms, ten echoes ranging from T.E. = 2.7 ms to 35.1 ms (echo spacing = 3.6 ms), and isotropic resolution of 60 × 60 × 60 μm^3^.

### 2.7. mRNA Sequencing

Six mice with a confirmed tumor of comparable size (as measured by bioluminescence imaging) were included in this experiment. Mice were divided into two groups (UMBO group and vehicle group). The vehicle group was treated with inVivoPure pH 6.5 Dilution Buffer (BXCELL, IP0065). Two treatment sessions (days 21 and 24) were applied in this experiment. Mice were sacrificed 24 h after the last treatment by cervical dislocation, and the right hemisphere was stored in 5 mL RNALater (Thermofisher AM7020). Lysing Matrix D (MBio, 6913050) was used to homogenize the collected brain tissues. mRNA was extracted using Maxwell RSC simply RNA automated RNA purification kit (Promega, AS1340). RNA quality was analyzed using high-sensitivity RNA chips. For RNA sequencing, NovaSeq 6000 sequencer (200 cycles, 800 million reads) and reagent kit. The reads (202 bp length, 100 million input reads) were mapped with the STAR v2.7.2a (default parameters) software to the reference genome (version GRCm38) on new junctions and known annotations. Mapping parameters were obtained from STAR outputs obtaining around 90% of unique mapped reads for all samples. Read counts from STAR were used as input for differential expression analysis using DESeq2. Furthermore, normalized counts were obtained using the variance-stabilizing transformation (VST) method from DESeq2 to be used as input for gene set variation analysis (GSVA) [21] to evaluate signature enrichment of microglia expression (Slc2a5, Siglech, P2ry12, Gpr34, P2ry13, Olfml3, Tmem119, and Fcrls) [22], microglia sensome (96 genes) [23] or antigen presentation related genes (Ciita, Psme2b, Erap1, Irf1, Tapbp, Psme2, Psme1, Pdia3, Psme3, Tap1, B2m, Calr, Tap2, Hspa1a, H2-Ab1k, H2-K1, and H2-D1) [24]. For heatmaps representation (ComplexHeatmap R package), VST gene expression values were first quantile normalized and log^2^ transformed, then converted to Z-scores by subtracting the average expression value of gene i (Gi) from the gene expression within sample x(Sx); the resulting value was divided by the SD of Gi; the formula is: Z-scoreGiSx = (Expression GiSx-μGi)/σGi. Raw data were uploaded to Gene Expression Omnibus with accession number GSE220909.

### 2.8. Immunohistochemistry (IHC)

A 150 KDa rat IgG2 antibody targeting PD-L1 was used in our IHC staining (BXCELL, #BE0101). A goat anti-rat secondary IgG (H+L) antibody (BA-9400) was used to detect the anti-PD-L1. Iba1 protein was detected using 1:1000 (Abcam, #ab178846). Mouse brains were fixed overnight in 4% paraformaldehyde (PFA), then immersed in 30% sucrose overnight for cryoprotection. Next, brains were stored in Tissue-Tek^®^ O.C.T and stored at −80 °C10 µm cryosections were harvested using Leica CM1950 cryostat. Slides were stored at −80 °C until analysis.

### 2.9. Quantitative Digital Droplet Polymerase Chain Reaction (ddPCR)

GL261 tumor-bearing mice four weeks following cell inoculation were used in the ddPCR experiment. A single UMBO treatment was completed, and 30 min later, blood (100 µL) was collected in heparinized tubes through cardiac puncture. Whole blood DNA was extracted automatically using Maxwell^®^ Blood DNA Purification Kit (AS1010). QX200 ddPCR EvaGeen^®^ was utilized to detect *mKate2* and *Luciferase* genes in the extracted DNA. Primer3Plus web interface was used to design *mKate2*, and *Luciferase* primers and primers were purchased from Life Technologies. The following forward (FR) and reverse (RV) primers were used: luciferase-FR, TCCACGATGAAGAAGTGCTC; *luciferase*-RV, AGGCTACAAACGCTCTCATC; *mKate2*-FR, GGTGAGCGAGCTGATTAAGG; and mKate2-RV, GGGTGTGGTTGATGAAGGTT.

### 2.10. Flow Cytometry

Twenty mice with a confirmed tumor of comparable sizes were included in this experiment. Mice were separated into four groups: UMBO group, anti-PD-L1 (Genentech, 6E11) group, UMBO plus anti-PD-L1, and vehicle group (*n* = 5/group). One treatment session was delivered in this experiment. Mice were perfused using cold distilled phosphate buffer saline (DPBS) ~16 h after treatment. Tumor-bearing hemispheres were isolated immediately and stored in 2 mL ice-cold Hanks’ balanced salt solution. According to the manufacturer’s protocol, the right hemisphere was isolated and mixed in the enzyme mix solution from the adult brain dissociation kit (Miltenyi Biotec, Cologne, Germany, #130-107-677). Cells gentleMACS^®^ Octo Dissociator with Heaters (#130-096-427) and gentleMACS C Tubes (#130-093-237) were used to perform mice brain dissociation. The number of dissociated cells was calculated using Scepter^®^ 3.0 Handheld Cell Counter. Flow cytometry was applied as described in Supplementary Material, Figure S2.

### 2.11. Statistical Tests

Statistical analysis was performed using Prism software (GraphPad Software, San Diego, CA, USA). Data are shown as mean values plus and minus standard error of the mean (SEM). Statistical significance of differences between groups was verified using appropriate statistical tests. Significance levels were denoted with asterisks: * for *p* ≤ 0.05; ** for *p* ≤ 0.01; *** for *p* ≤ 0.001, and **** for *p* ≤ 0.0001.

## 3. Results

### 3.1. Repeated UMBO Is Safe and Effective in Immunocompetent Mice

**Figure 2:**
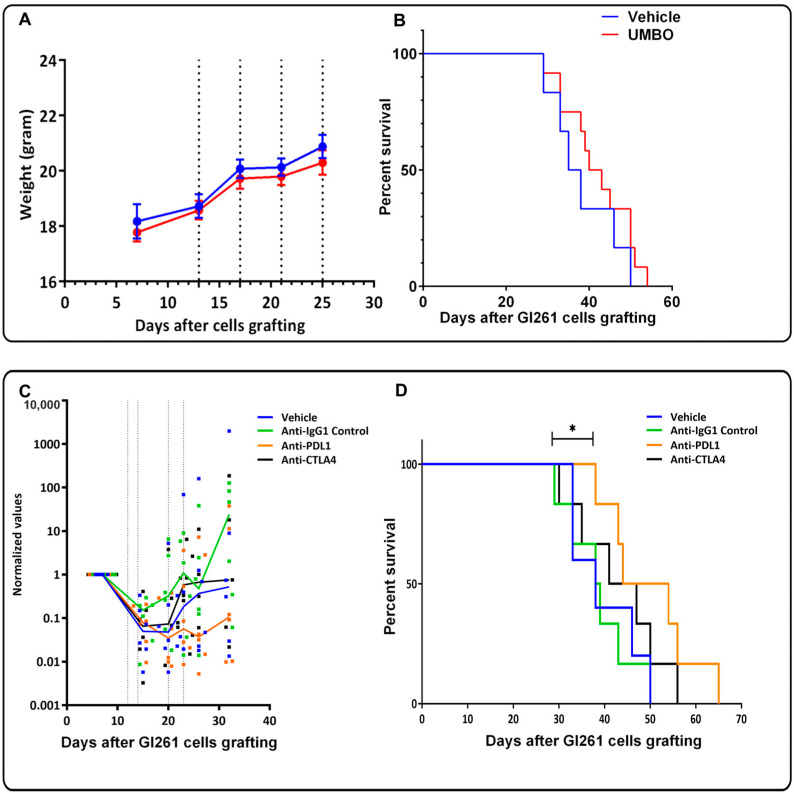
UMBO parameters are safe and anti-PD-L1 increased survival of GL261 bearing mice. Repeated UMBO alone does not affect OS (Panel **B**) or body weight in GL261-bearing mice compared to the non-treated (Panel **A**). (Panel **C**,**D**): Animals were treated with 200 µg of anti-PDL-1, Anti-IgG1 as (a control), anti-CTL-4, or vehicle for four doses. Bioluminescence measures normalized to the first measured value performed on day 7 after cell inoculation in GL261-bearing mice model. Each colored dot represents values for one animal and the line represents the median value for the group. Bioluminescence signal was measured weekly. Dotted lines represent the days of treatments. (Panel **D**). Kaplan Meier curves in GL261-bearing mice. Anti-PD-L1 alone improved OS in Gl261 bearing mice. Anti-CTLA-4 did not improve OS in GL261-bearing mice. * for *p* ≤ 0.05.

UMBO parameters were previously optimized [25] in healthy mice and we evaluated UMBO parameters and treatment frequency in GL261 bearing mice. T_1_w MRI (Figure 1C) showed a marked gadolinium contrast enhancement within an hour following the UMBO (Figure 1C). Furthermore, biweekly UMBO (four sonications in total without drugs) was evaluated in the GL261-bearing mice. Mouse weight was unaffected (Figure 2A) and no significant difference in the overall survival (OS) between UMBO and non-treated groups was observed (Figure 2B). Overall, the UMBO parameters used for repeated BBB opening were safe and well-tolerated in GL261-bearing mice.

Pilot study with no UMBO using a GL261-luciferase orthotopic GBM mouse model was performed. This experiment aimed to determine the effect of anti-PD-L1 and anti-CTLA-4 in our GBM mouse model and select the best candidates to combine with UMBO. Anti-PD-L1 antibody alone showed a small regression in tumor growth (Figure 2C) with a limited improvement of survival in GL261-bearing mice (Figure 2D), Anti-CTLA-4 treatment did not reduce tumor growth (Figure 2C) or animal survival (Figure 2D).

Furthermore, using immunofluorescence, we were able to detect PD-L1 expression on the surface of the GL261 cell line using anti-PD-L1 (Genentech, 6E11). RT-PCR was used to evaluate the quantitative expression of PD-L1 in the GL261 cell line and we have used the Nfpp10 cell line as a positive control. PD-L1 expression was significantly higher in the GL261 cell line compared to our control (Supplementary Material, Figure S3). Overall, this makes the anti-PD-L1 antibody the best candidate to combine UMBO in the GL261 GBM mouse model.

### 3.2. UMBO Dramatically Increased the Efficacy of anti-PD-L1 in GL261-Bearing Mice

**Figure 3:**
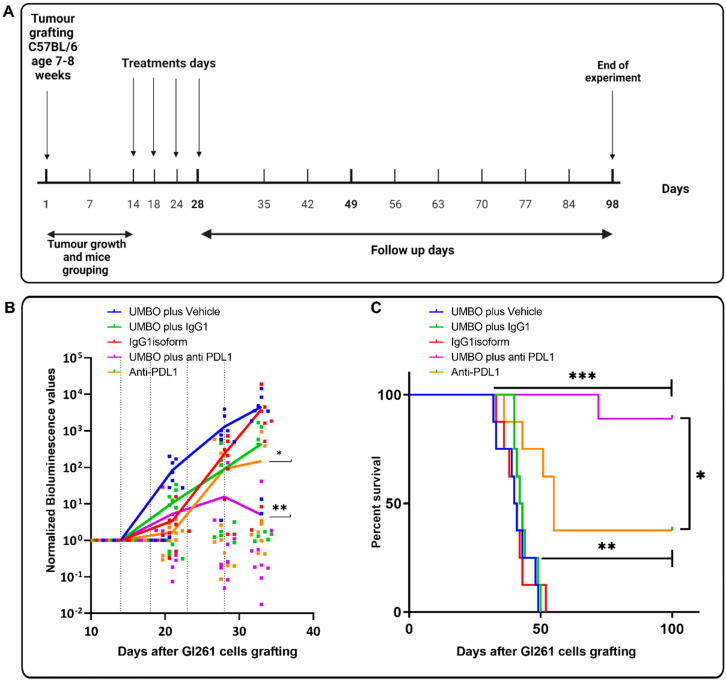
UMBO increased the efficacy of anti-PD-L1 in GL261-bearing mice. (Panel **A**): shows a timeline of the treatment protocol used in the survival experiments. Cell grafting was done on day 1 while treatments were applied on days 14, 18, 24, and 28. Mice were followed up for 100 days. (Panel **B**,**C**): (Panel **B**): bioluminescence normalized to the first measured value after cell inoculation in GL261-bearing mice. Each dot represents values for one animal and the line represents the median value for the group. Bioluminescence signal was measured weekly. Dotted lines represent the days of treatments. Anti PD-L1 plus UMBO suppressed tumor growth over time compared to controls. ** for *p* ≤ 0.01. Repeated UMBO alone does not affect OS (Panel **C**) or body weight (Supplementary Material, Figure S4) in GL261-bearing mice compared to the non-treated (Panel **C**). (Panel **C**): UMBO plus an-ti-PD-L1 increased OS (* *p* < 0.05) compared to anti-PD-L1 alone and (*** *p* < 0.0001) compared to control groups.

We then investigated the combined effect of UMBO with anti-PD-L1 in the GL261-bearing mice. Mice with comparable bioluminescence values were divided into five groups: (i) UMBO group, (ii) anti-PD-L1 group, (iii) UMBO plus anti-PD-L1 group, (iv) IgG1 group, and (v) IgG1 plus UMBO group. Mice that had received an anti-PD-L1 antibody plus UMBO showed 76% long-term survivals (13/17) compared to 26% in anti-PD-L1 alone (4/15) and 0% in control groups (0/16). The results from two independent experiments (Exp) were summarized in Table 1. UMBO plus anti-PD-L1 showed a significant regression in tumor growth following four sessions of treatments compared to controls (*p* < 0.01) (Figure 3B). Furthermore, Anti-PD-L1 alone showed a limited regression of tumor growth (*p* < 0.05) compared to control (Figure 3B). Kaplan–Meier estimate showed a significant difference in OS of UMBO’s plus anti-PD-L1 treated mice versus anti-PD-L1 alone (Figure 3C). Furthermore, a higher significant difference (Figure 3C) was observed in UMBO plus anti-PD-L1 treated mice compared to anti-IgG1 plus UMBO treated mice.

### 3.3. UMBO Increased the Penetration of anti-PD-1 and anti-PD-L1 Antibodies into the Brain Parenchyma

**Figure 4:**
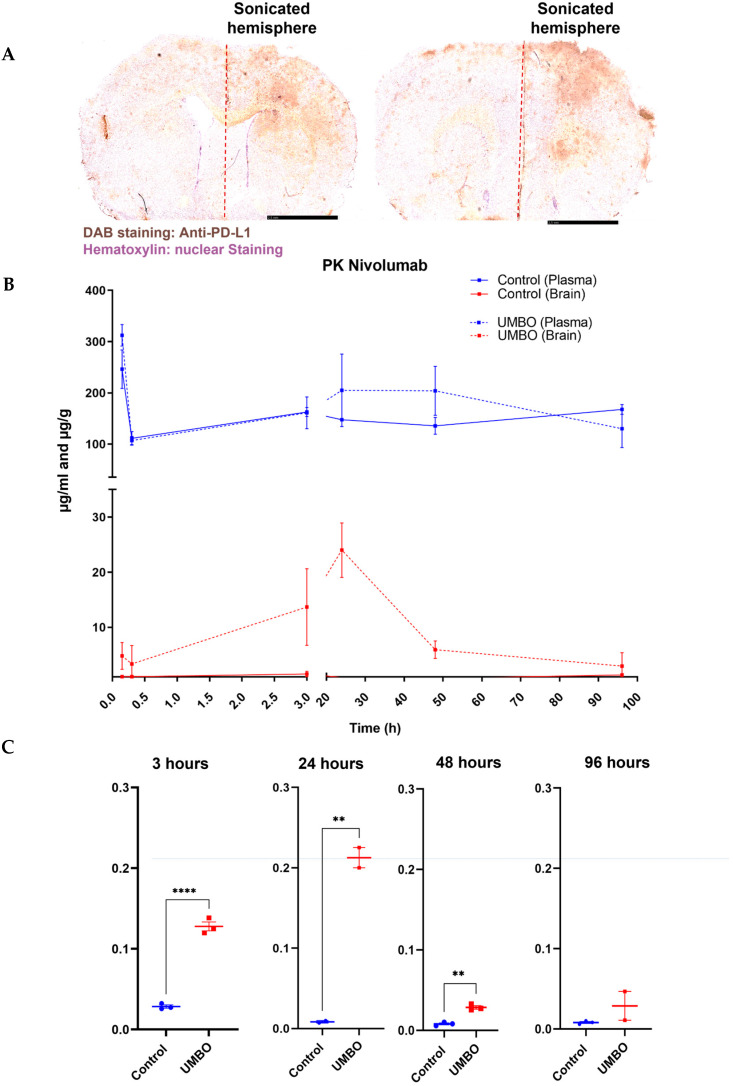
UMBO increased the delivery of ICIs to the brain parenchyma. (Panel **A**): IHC staining of anti-PD-L1 (BXCELL) (Panel **B**): PK of Nivolumab concentration in the C57BL/6 mice blood and brain. (Panel **C**): Brain/plasma ratio of nivolumab concentration over time. UMBO enhanced the brain/plasma ratio of nivolumab compared to control mice. ** for *p* ≤ 0.01. **** for *p* ≤ 0.0001.

IHC staining of anti-PD-L1 (BXCELL, BE0101) confirmed UMBO’s trend to deliver anti-PD-L1 to the right hemisphere brain parenchyma (Figure 4A). Furthermore, an already clinically optimized method [21] was used to compare a size matched IgG1 antibody’s pharmacokinetics with and without UMBO. Three C57BL/6 mice per time point (six-time points) per group were used in the analysis. We observed a comparable serum concentration of nivolumab in control and UMBO-treated mice.

Interestingly, higher concentrations of nivolumab were detected in mice brains treated with nivolumab plus UMBO. As expected, we detected a negligible concentration (≤0.2 µg/200 mg brain) of nivolumab in control mice brains (Figure 4B). The maximum concentration (*C*_max_) of nivolumab in normal brain tissue was detected at 24 h and started to decline and reach a negligible concentration at 96 h. Therefore, a regimen of biweekly antibody administration was performed. The brain to plasma concentration ratio shows that UMBO enhanced the ratio of nivolumab passage across the BBB at 3, 24, 48 h but not at 96 h (Figure 4C).

### 3.4. UMBO plus Anti-PD-L1 Activates Microglia and Modulates Microglial Phenotype

**Figure 5:**
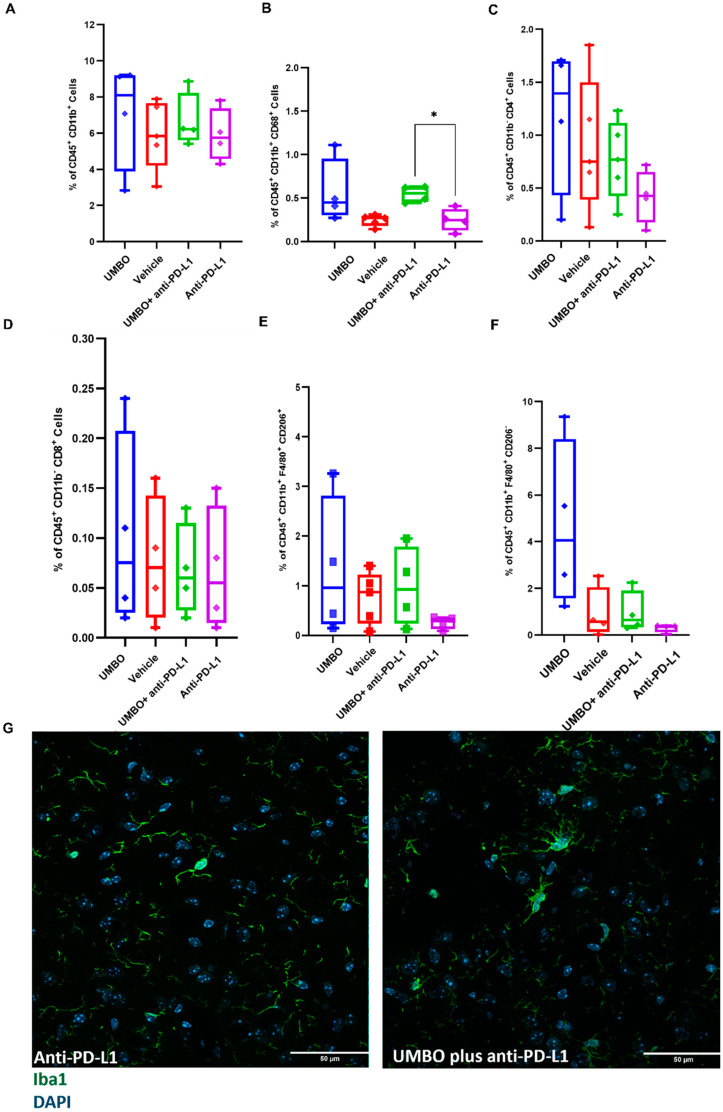
UMBO plus anti-PD-L1 activates microglia and modulates microglial phenotype. (Panel **A**): Flow cytometry analysis of the percentage of total microglia in all different groups (*n* = 4–5). (Panel **B**): UMBO plus anti-PD-L1 significantly enhanced (* *p* < 0.05) the percentage of CD68^+^ cells than an-ti-PD-L1 alone. (Panel **C**,**D**): UMBO plus anti-PD-L1 did not influence CD4^+^ and CD8^+^ T-lymphocytes percentages compared to other groups. (Panel **E**,**F**): No significant difference in CD206^+^ and CD206^−^ macrophages in all groups. UMBO plus anti-PD-L1 did not modulate macrophages’ expression. (Panel **G**): Green: Iba1 microglia/macrophages Blue: DAPI nuclear staining; microglia staining in anti-PD-L1 plus UMBO (right photos) treated group confirm a phenotype of activated microglia.

We studied the immune cell populations in our treatment groups using flow cytometry. Interestingly, we observed that UMBO plus anti-PD-L1 significantly enhanced the percentage of activated microglia compared to anti-PD-L1 treatment alone (Figure 5B). UMBO alone was not associated with a significant enhancement of activated microglia percentage compared to the vehicle group; however, a trend was observed (*p* = 0.150). On the other hand, we did not observe any significant changes in the percentage of CD8^+^ and CD4^+^ T-lymphocytes or CD206^+^ macrophages in all groups. Immunofluorescence staining of microglia in the anti-PD-L1 plus UMBO treated group confirmed this finding and showed a phenotype of activated microglia. IHC showed a double nucleus staining of Iba1 in the UMBO plus anti-PD-L1 treated GL261-bearing mice suggesting a possible induction of microglia cell division (Figure 5G).

**Figure 6:**
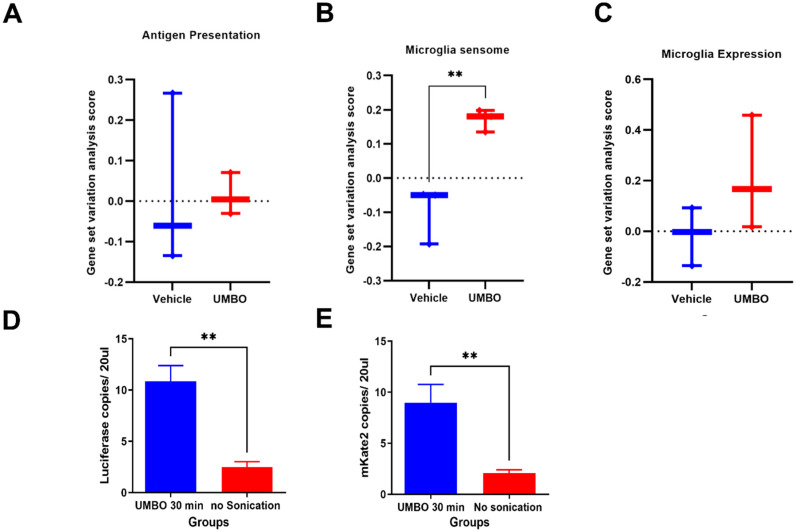
Repeated UMBO is associated with microglia’s gene signature in the GL261 mouse model. (Panel **A**): UMBO did not influence antigen presentation gene signature compared to the non-treated. (Panel **C**): UMBO alone is associated with a significant (** *p* < 0.01) enrichment of microglia sensome gene signature (Panel **D**) compared to vehicle (*n* = 3). No significant difference was observed in microglial gene expression (Panel **B**). Heat maps of microglia gene signature and antigen presentation genes used in panels A and C are presented in Supplementary Material, Figure S5. (Panel **D**,**E**) represent the ddPCR analysis of *Luciferase* DNA (Panel **D**) and *mKate2* DNA (Panel **E**) in the blood 30 min following UMBO.

Using bulk RNA sequencing, we analyzed whether UMBO modulates antigen presentation related genes compared to the vehicle treated group. UMBO did not influence antigen presentation (Figure 6A) or affect microglia gene expression (Figure 6B). Microglial ability to sense changes in the cellular environment was recently termed as microglia sensome [23]. We used the same gene signature to evaluate microglial sensome with and without UMBO. Interestingly, UMBO significantly induced the expression of gene signatures for microglial sensome compared to control (Figure 6B). Additionally, we evaluated whether UMBO enhanced circulating tumor DNA release to the bloodstream. GL261-bearing mice with significant tumors were used in this experiment. We observed a significant elevation in the number of copies for both *luciferase* (Figure 6D) and *mKate2* (Figure 6E) in the UMBO treated group compared to the control. 

## 4. Discussion

UMBO and several innovative strategies continuously evolve to overcome the BBB by increasing drug delivery [8]. Immunotherapies, including ICIs and cell therapies, have revolutionized multiple solid tumors’ treatments through activating the general antitumor immune response. The CheckMate-143 phase three clinical trial failed to demonstrate any higher efficacy of nivolumab over bevacizumab and showed that nivolumab did not improve OS in GBM patients. Several reasons might explain the low efficacy of ICIs in GBM: (i) low tumor mutation load, (ii) lack of predictor of response and lack of selection of patients, (iii) low penetration of ICIs within the brain parenchyma, (iv) low peripheral priming, (v) local immunosuppression, and (vi) low penetration of T-lymphocytes [26].

We explored the BBB as the limitation for antibody and lymphocytes penetration and priming and evaluated the potential of UMBO to deliver large therapeutics to the brain and the possible modulation of the immune microenvironment in GBM mouse models. We confirmed the limited efficacy of ICIs in the Gl261-bearing and Nfpp10-bearing mouse models. Consistent with our data, Reardon et al. reported limited efficacy of anti-PD-L1 and anti-CTLA-4 in GL261-bearing mice with a different treatment regimen [27].

To the best of our knowledge, this is the first research article that reports a dramatic increase in the OS of GL261-bearing mice when treated with UMBO plus anti-PD-L1. Indeed, 76% of GL261-bearing mice treated with anti-PD-L1 plus UMBO survived longer than 100 days compared to 26% of mice treated with anti-PD-L1 alone. We investigated the mechanisms involved in the anti-tumor effect. We hypothesized that the BBB was responsible for the limited efficacy by blocking anti-CTLA-4 and anti-PD-L1 from reaching the GBM tumor. A recent study reported enhanced efficacy of antibody conjugates following their delivery to GBM tumors [28]. Furthermore, focused ultrasound enhanced the delivery of intranasal anti-PD-L1 but not OS in GL261-bearning mice [29] and a very recent clinical study showed that UMBO enhanced the delivery of trastuzumab in brain metastases [30].

In our setting, we reported that UMBO enhanced antibody concentration up to 28-fold compared to control. UMBO was optimized to disturb one hemisphere; however, in our PK analysis, we used a whole-brain homogenization method; therefore, local concentrations of nivolumab could have been higher. Consistent with our data, a study has shown that UMBO enhanced the delivery of bevacizumab [31] ~149 KDa and anti-PD1 [32] compared to non-sonicated brains in a glioma mouse model. UMBO plus 200 µg of the anti-PD-L1 biweekly treatment regimen was used to maintain the higher concentration of anti-PD-L1 within the brain parenchyma. Immune checkpoint blockade with anti-PD-L1 was performed on day 14 post-inoculation to allow for T-lymphocytes exhaustion [33].

UMBO stimulates detectable peripheral circulation of GL261 DNA [34]. Zhu et al., investigated the possibility of using UMBO for liquid biopsies in GBM models. They detected that green fluorescent protein mRNA 20 minutes following UMBO in the GL261-GFP expressing mouse model [35] supports the passage of tumor material from the brain to blood flow stream. Consistent with our timepoint, another research group described that UMBO increases cell-free DNA in time-dependent matter [36]. The priming effect of circulating DNA could activate naïve T-lymphocytes through their exposure to new antigens. BBB protects the tumor from T-lymphocytes infiltration and immune activation. Therefore, detecting GL261 tumors in the peripheral circulation might activate the global antitumor effect. Further functional analysis of lymphocyte activation should be performed to evaluate any priming effect of UMBO.

Our results showing microglia activation in the UMBO plus anti-PD-L1 treated GL261-bearing mice suggest a possible mechanism for the observed enhanced therapeutic efficacy of anti-PD-L1. Our flow cytometry analysis confirms published data that showed a higher ratio of Iba-1 staining in sonicated regions compared to the non-sonicated [37]. PD-L1 is expressed on the cell surface of both GL261 and microglia [38]. A possible effect on microglia phenotype might be related to the combined effect of UMBO and anti-PD-L1 delivery to the brain parenchyma. Activated microglia might have a cytotoxic effect against GL261 tumor cells [39]; therefore, further investigation of the activated microglia role in GBM should be addressed.

To date, there is no clear evidence of the effect of UMBO on T-lymphocytes passage to the brain. We did not observe any significant elevation in the percentage of CD8^+^ and CD4^+^ T-lymphocytes at one timepoint (~16 h). This effect might be related to the timing of sample collection as we only evaluated our treatment regimen at one timepoint. We observed a delayed antitumor effect in UMBO and anti-PD-L1 group which can be related to a delayed effect on T-lymphocytes. Furthermore, we have not analyzed any subpopulations of CD8^+^ T lymphocytes i.e., PD-1^+^ CD8^+^ T-lymphocytes.

Syngeneic mice models and especially the GL261 mouse model used in our experiments is a limitation of the current study. The GL261 mouse model: (i) has a high mutation load which is not consistent with GBM patients and (ii) a variability in terms of responses to ICIs in vivo [33]. Another limitation is the inability to demonstrate functional analysis of the role of UMBO in priming naïve T-lymphocytes through their exposure to new antigens. Additional functional analysis of the effect of UMBO plus anti-PD-L1 would explain the dramatic effect on OS that was observed in our study.

## 5. Conclusions

Our study showed statistically significant increased brain penetration and efficacy of anti-PD-L1 in GL261-bearing mice when delivered by UMBO. We have also provided clear evidence of the possible safe and effective delivery of large therapeutic agents using UMBO. Further investigations are needed to confirm the impact of UMBO on brain penetration and efficacy of chemotherapeutic agents and anti-PD-L1 to overcome the resistance of GBM to the current treatments.

## Figures and Tables

**Table 1:** Summary of long-term survivals and statistical analyses.

		Exp. 1	Exp. 2
No of Long-term Survivals > 100 days	UMBO plus vehicle	0/8	0/8
	UMBO plus IgG1	0/8	0/8
	IgG1 isoform	0/8	0/8
	UMBO plus anti-PD-L1	7/9	6/8
	Anti-PD-L1	3/8	1/7
Percentage	UMBO plus anti-PD-L1	77%	75%
	Anti-PD-L1	37%	14%
Statistic	UMBO plus anti-PD-L1/UMBO alone	*p* = 0.0009	*p* = 0.0012
	anti-PD-L1/IgG1 isoform	*p* = 0.0057	*p* = 0.0191
	UMBO plus anti PD-L1/anti-PD-L1	*p* = 0.0480	*p* = 0.0360

## Data Availability

Raw data will be available upon request from the authors.

## Supplementary Materials

The following supporting information can be downloaded at: https://www.mdpi.com/article/10.3390/pharmaceutics15020455/s1, Figure S1: Schematic representation of LIPU procedure. (A) Graphical representation of LIPU generator set up. (B) Experimental timeline for anti-PD-1 treatment with BBB disruption. (C) A quantitative analysis of Gd enhancement pre and post UMBO in Gl261-bearing mice. (** *p* < 0.001); Figure S2: A representative gating strategy to identify immune cell subsets in Gl261 bearing brains following treatments. Mice were perfused using cold distilled phosphate buffer saline (DPBS) ~16 h after treatment. Tumor-bearing hemispheres were isolated, dissociated. Samples were acquired on a spectral flow cytometer (Aurora, Cytek) and analyzed by FlowJo software (FlowJo, LLC). Briefly, cells were selected based on their morphology, doublets, and dead cells were excluded using (Biolegend, #423107) while tumor cells were excluded based on their *mKate* expression. Monocytes (Ly6C^+^ Ly6G^−^) and neutrophils (Ly6C^+^ Ly6G^+^) were excluded from non-tumoral live cells using Ly-6C (Biolegend, #128036) and Ly-6G (Biolegend, #127617). Microglia were identified based on their expression of CD11b^+^ and CD45^low^ using CD45 (Biolegend, #103131) and CD11b (Biolegend, #101255). Activated microglia were identified as CD68^+^ using (Biolegend, #137003). F4/80 marker (Biolegend, #123117) was used to determine macrophages in the CD45^high^ CD11b^+^ cell population. CD206 marker (Biolegend, #141729) was used to distinguish between subpopulations of macrophages. Lymphocytes CD4^+^ (Biolegend, #100541) and CD8^+^ (Biolegend, #100737) were identified on the CD45^+^ CD11b^−^ fraction of non-tumoral live cells. The percentage of each subpopulation was calculated and used for comparisons; Figure S3: Quantitative expression of PD-L1 in GL261 cell line and we have used Nfpp10 cell line as a positive control (left figure). Immunofluorescence of PD-L1 expression in GL261 cell line. (* *p* < 0.05); Figure S4: None of the treatments affected the body weight of GL261 bearing mice. Analysis of body weight shows no significant changes on body weight following treatments. Dotted lines represent the days of treatments; Figure S5: Heat maps of microglia gene signature (left figure) and antigen presentation genes (right figure) used in data analysis in Figure 6A,C.
