# Supplementary material for: Low-Intensity Pulsed Ultrasound-Mediated Blood-Brain Barrier Opening Increases Anti-Programmed Death-Ligand 1 Delivery and Efficacy in Gl261 Mouse Model
Source: Pharmaceutics. 2023 Jan 30;15(2):455. doi: 10.3390/pharmaceutics15020455 (PMC9967384; doi:10.3390/pharmaceutics15020455)
Supplement: Supplementary file 1 [file pharmaceutics-15-00455-s001.zip › pharmaceutics-2116866-supplementary.pdf]

pharmaceutics

MDPI

Supplementary Materials: Low-Intensity Pulsed Ultrasound-Mediated Blood-Brain Barrier Opening Increases Anti-Programmed Death-Ligand 1 Delivery and Efficacy in Gl261 Mouse Model

Mohammed H Ahmed, Isaias Hernández-Verdin, Emie Quissac, Nolwenn Lemaire, Coralie Guerin, Lea Guyonnet, Noël Zahr, Laura Mouton, Mathieu Santin, Alexandra Petiet, Charlotte Schmitt, Guillaume Bouchoux, Michael Canney, Marc Sanson, Maïté Verreault, Alexandre Carpentier and Ahmed Idbaih

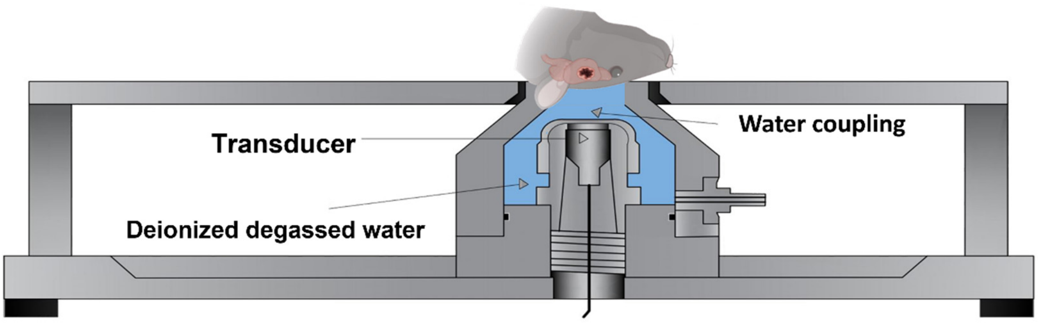

(**A**)

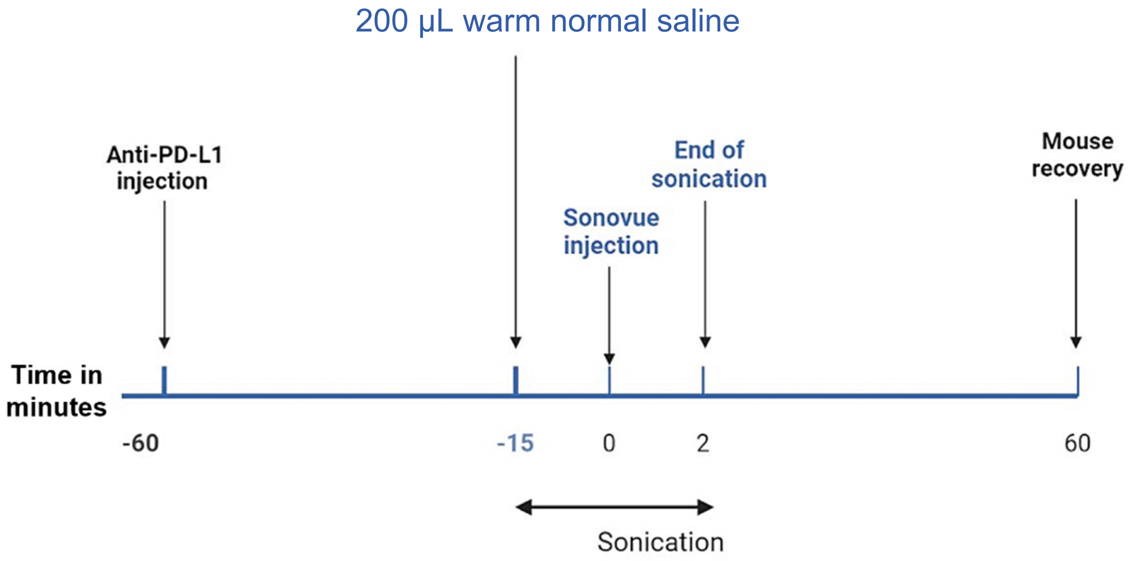

(**B**)

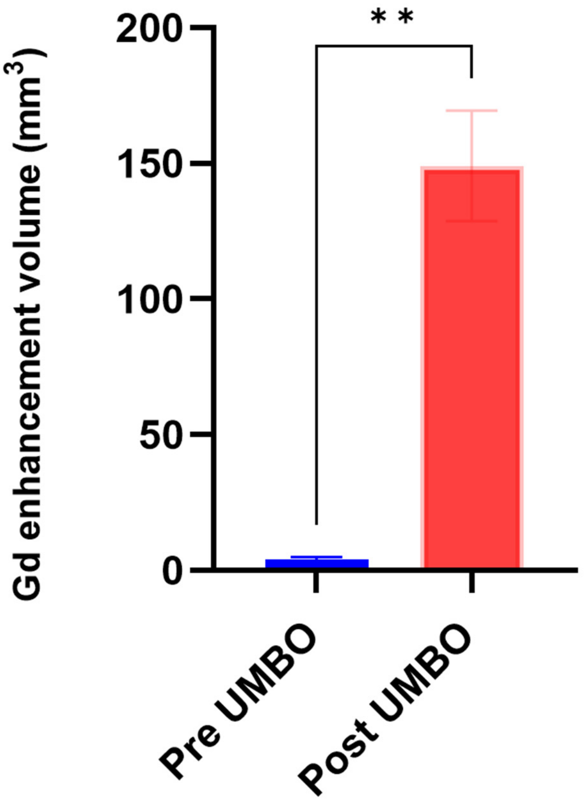

(**C**)

Figure S1. Schematic representation of LIPU procedure. (**A**) Graphical representation of LIPU generator set up. (**B**) Experimental timeline for anti-PD-1 treatment with BBB disruption. (**C**) A quantitative analysis of Gd enhancement pre and post UMBO in Gl261-bearing mice. (** $p < 0.001$).

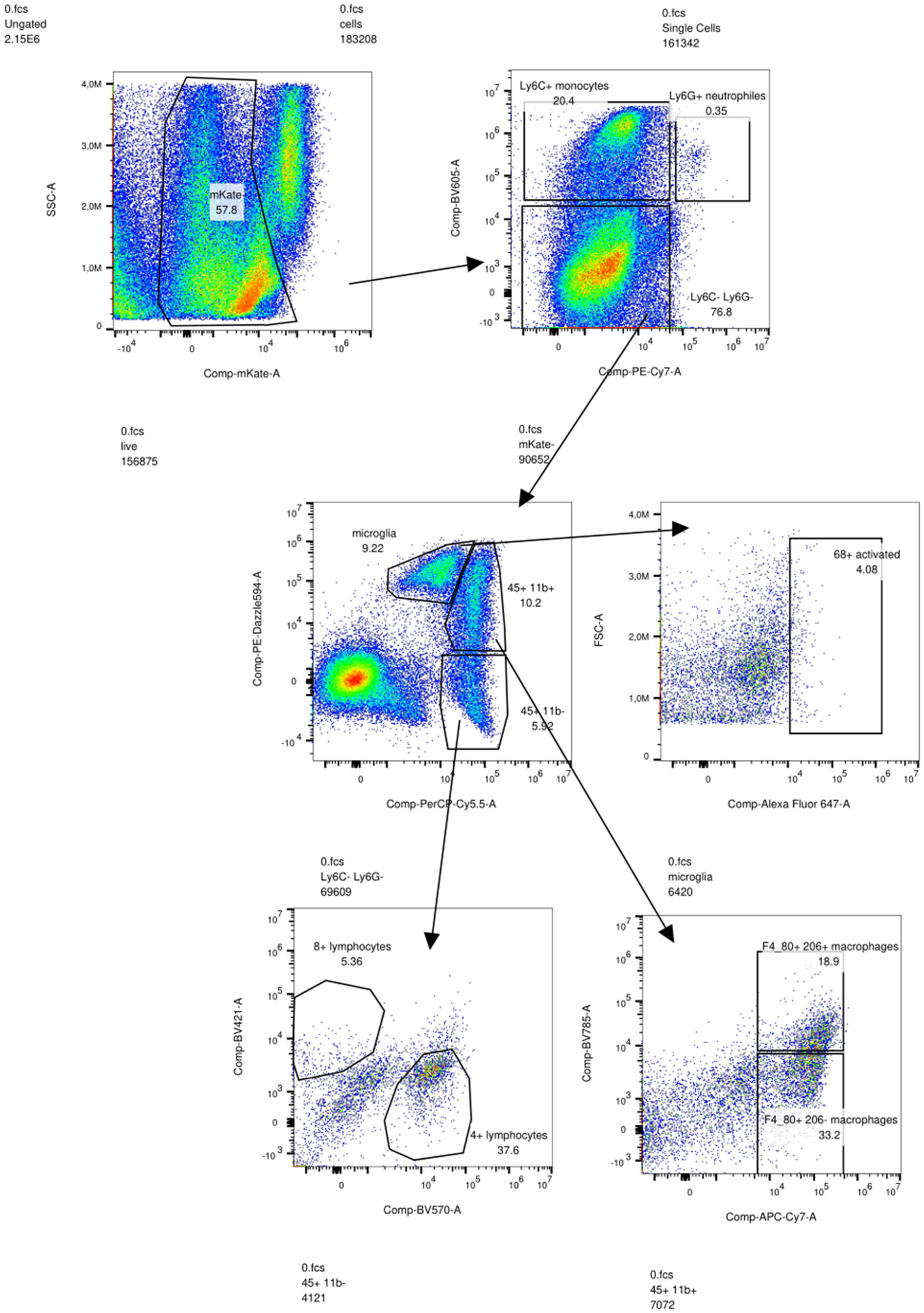

Figure S2. A representative gating strategy to identify immune cell subsets in Gl261 bearing brains following treatments. Mice were perfused using cold distilled phosphate buffer saline (DPBS) ~16 h after treatment. Tumor-bearing hemispheres were isolated, dissociated. Samples were acquired on a spectral flow cytometer (Aurora, Cytek) and analyzed by FlowJo software (FlowJo, LLC). Briefly, cells were selected based on their morphology, doublets, and dead cells were excluded using

(Biolegend, #423107) while tumor cells were excluded based on their *mKate* expression. Monocytes (Ly6C$^+$ Ly6G$^-$) and neutrophils (Ly6C$^+$ Ly6G$^+$) were excluded from non-tumoral live cells using Ly-6C (Biolegend, #128036) and Ly-6G (Biolegend, #127617). Microglia were identified based on their expression of CD11b$^+$ and CD45^{low} using CD45 (Biolegend, #103131) and CD11b (Biolegend, #101255). Activated microglia were identified as CD68$^+$ using (Biolegend, #137003). F4/80 marker (Biolegend, #123117) was used to determine macrophages in the CD45^{high} CD11b$^+$ cell population. CD206 marker (Biolegend, #141729) was used to distinguish between subpopulations of macrophages. Lymphocytes CD4$^+$ (Biolegend, #100541) and CD8$^+$ (Biolegend, #100737) were identified on the CD45$^+$ CD11b$^-$ fraction of non-tumoral live cells. The percentage of each subpopulation was calculated and used for comparisons.

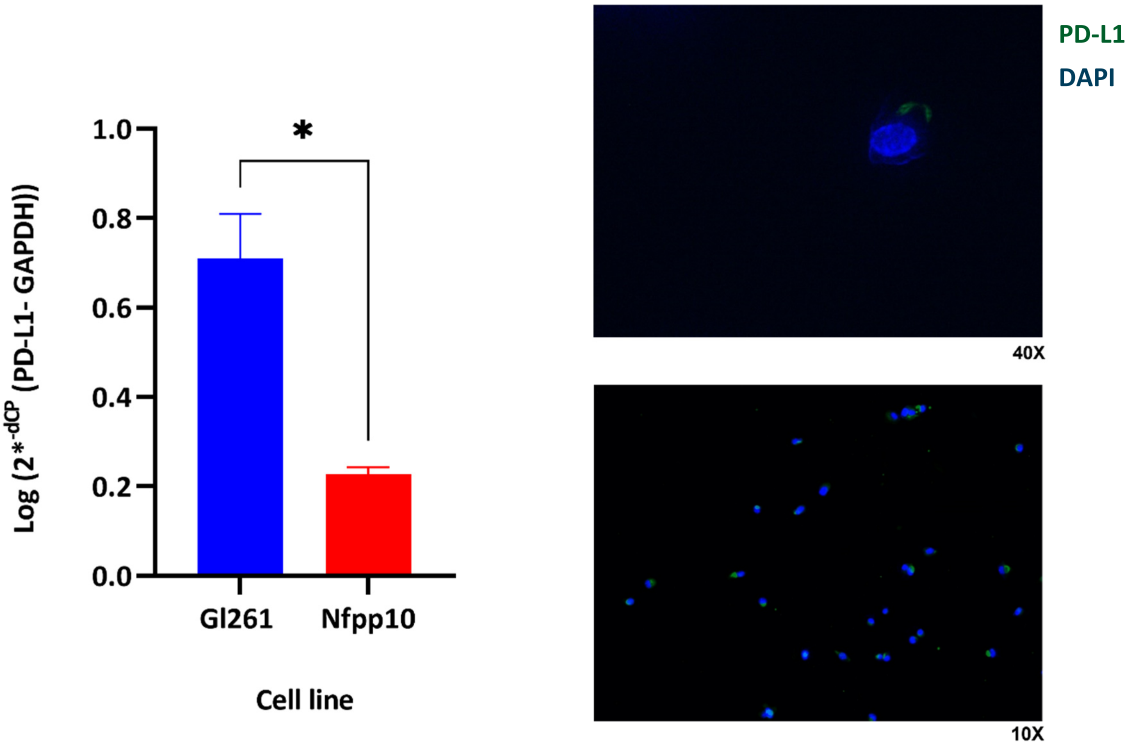

Figure S3. Quantitative expression of PD-L1 in GL261 cell line and we have used Nfpp10 cell line as a positive control (left figure). Immunofluorescence of PD-L1 expression in GL261 cell line. (* $p < 0.05$).

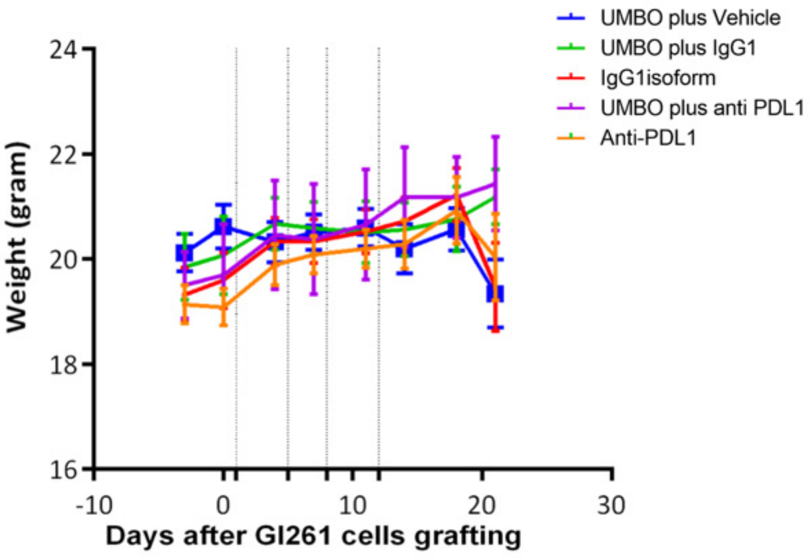

Figure S4. None of the treatments affected the body weight of GL261 bearing mice. Analysis of body weight shows no significant changes on body weight following treatments. Dotted lines represent the days of treatments.

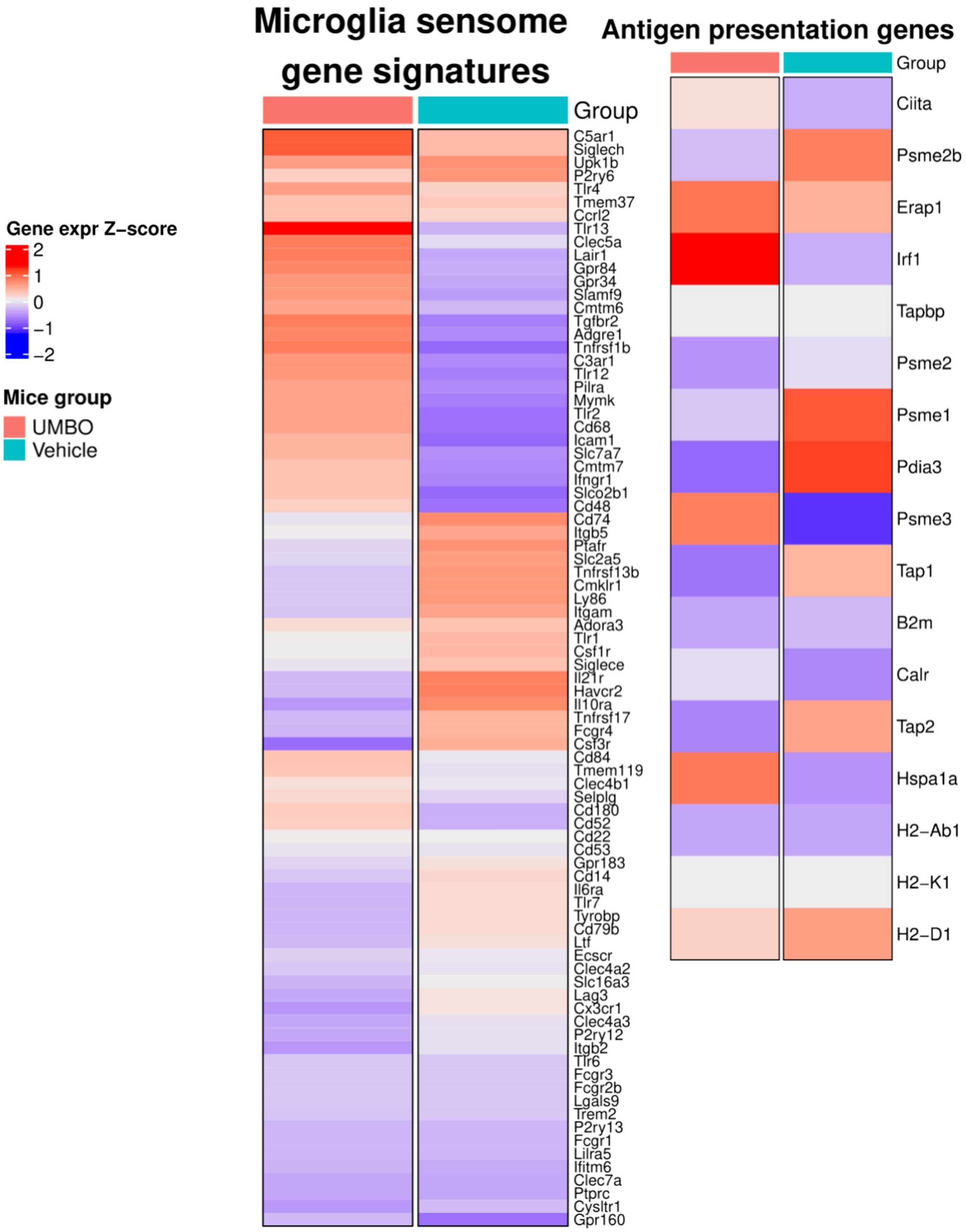

Figure S5. Heat maps of microglia gene signature (left figure) and antigen presentation genes (right figure) used in data analysis in Figure 6 panels **A** and **C**.
